# Correction: COVID-19 vaccination in Sindh Province, Pakistan: A modelling study of health impact and cost-effectiveness

**DOI:** 10.1371/journal.pmed.1003990

**Published:** 2022-05-04

**Authors:** Carl A. B Pearson, Fiammetta Bozzani, Simon R. Procter, Nicholas G. Davies, Maryam Huda, Henning Tarp Jensen, Marcus Keogh-Brown, Muhammad Khalid, Sedona Sweeney, Sergio Torres-Rueda, CHiL COVID-19 Working Group, CMMID COVID-19 Working Group, Rosalind M. Eggo, Anna Vassall, Mark Jit

Reference item 35 cites the incorrect version of WHO guidance. The correct reference is: Initiative for Vaccine Research. WHO guide for standardization of economic evaluations of immunization programmes [Internet]. WHO; 2019 [cited 2021 Jan 15]. Available from: https://apps.who.int/iris/bitstream/handle/10665/329389/WHO-IVB-19.10-eng.pdf.
