# Supplementary material for: COVID-19 vaccination in Sindh Province, Pakistan: A modelling study of health impact and cost-effectiveness
Source: PLoS Med. 2021 Oct 4;18(10):e1003815. doi: 10.1371/journal.pmed.1003815 (PMC8523052; doi:10.1371/journal.pmed.1003815)
Supplement: Attachment — Submitted filename: PLoS Med Response to Reviewers.pdf [file pmed.1003815.s002.pdf]

Please see below for our responses to items from the editors and reviewers. Our responses are in blue. Where we are including revised text from the manuscript, we have indented and italicized that text.

Requests from the editors

1. Please revise your title according to PLOS Medicine's style. Your title must be nondeclarative and not a question. It should begin with main concept if possible. Please place the study design (for example, "A cost-effectiveness analysis", "A modelling study") in the subtitle (i.e., after a colon).

We have altered the title to "COVID-19 vaccination in Sindh Province, Pakistan: a modelling study of health impact and cost-effectiveness".

2. Abstract summary - At this stage, we ask that you reformat your non-technical Author Summary. The Author Summary should immediately follow the Abstract in your revised manuscript. This text is subject to editorial change and should be distinct from the scientific abstract. The summary should be accessible to a wide audience that includes both scientists and non-scientists. Please see our author guidelines for more information: https://journals.plos.org/plosmedicine/s/revising-your-manuscript#loc-author-summary.

We have revised the "Research in Context" into an "Author Summary" section, as per the guidelines.

3. Abstract:
a. Please structure your abstract using the PLOS Medicine headings (Background, Methods and Findings, Conclusions).
b. Please combine the Methods and Findings sections into one section, "Methods and findings". Please ensure that all numbers presented in the abstract are present and identical to numbers presented in the main manuscript text.
c. In the abstract, please include the important parameters included in your model.
d. In the last sentence of the Abstract Methods and Findings section, please describe the main limitation(s) of the study's methodology.

a. We have revised the Abstract to comply with this structure.
b. We have combined these sections, and checked all numbers presented against the main text.
c. Our model includes many parameters, but we now particularly highlight the values of the most important (vaccine cost, vaccine efficacy, duration of protection).
d. We now mention the following limitations in the abstract:

These projections are limited by the mechanisms present in the model. Because the model is a single-population compartmental model, detailed impacts of non-pharmaceutical interventions (NPIs) such as household isolation cannot be practically represented or evaluated in combination with vaccine programmes. Similarly, the model cannot consider prioritizing groups like healthcare or other essential workers. Additionally, because the future impact and implementation cost of NPIs is uncertain, how these would interact with vaccination remains an open question.

4. Please avoid assertions of primacy ("Our study provides the first combined epidemiological and economic analysis ...."). Instead use the phrase, “To our knowledge, this is the first…”

We have removed this assertion from what is now the “Author Summary”.

5. Please use the "Vancouver" style for reference formatting, and see our website for other reference guidelines
https://journals.plos.org/plosmedicine/s/submission-guidelines#loc-references.

We have revised the reference format to the Vancouver style as well as ensuring that other formatting requirements are met.

Reviewer #1

General Comments

This manuscript contributes to the evidence base on the cost-effectiveness of COVID-19 vaccination relative to no vaccination in low- and middle-income countries. Within a constrained vaccine supply environment, the authors set out to determine the most cost-effective vaccine prioritisation strategy for LMICs. To do this they combined an epidemiological and economic model to assess the health impact, economic impact and cost-effectiveness of Covid-19 vaccination in the Sindh province, Pakistan. The authors show that it is still cost-effective to vaccinate a very small proportion of the population regardless of age-related targeting strategy as long as the vaccine is reasonable priced, efficacy is high, and a reasonable period of natural- and vaccine-induced immunity. This paper provides compelling evidence for the cost-effectiveness of a small-scale COVID-19 vaccination programme irrespective of targeting strategy.

Minor Essential Revisions

*** Vaccine distribution. What does a vaccine campaign of 5 or 10 years mean? Are people vaccinated continuously over the period at a rate of 4000 vaccines/day? Is it assumed that a different segment of the population is vaccinated each year? On page 18, the authors state that "Administering 4000 doses/day in a province of roughly 50**

million people would need to be continued for a long time for vaccination to have a large impact". What do the authors mean by 'large impact' - greater reduction in cases and mortality, or closer to herd immunity? At a rate of 4000 doses/day, after 1 year approximately 1.5million (out of a population of 50 million people) will be vaccinated. This is a very small proportion of the population (3%). It would be interesting for the authors to show a scenario that involves a different roll-out from that predicted based on COVAX but results in the ability to vaccinate a higher proportion of the population (that would be closer to a herd immunity target) in one year. Would this still be cost-effective?

*** Rate at which vaccine roll-out occurs. The authors also state that "For simplicity, vaccination occurs at the same rate on every day in the model" and that the "slow rate of vaccine distribution is the major impediment to larger health impact" (page 18). How would changing the rate change the results? How would a slower-rate of roll-out affect the results?**

Thank you - we agree these points need clarification. We use the phrase “campaign” to distinguish from activities like routine childhood immunization; campaign-style programmes have different costs. We have modified the manuscript to avoid the word “campaign” until explaining that usage with this new text in the “Methods: Costs” subsection:

> *It was assumed that all vaccine doses would be delivered through campaigns in the community.*

Relative to courses delivered per day, we do not assume only 4000 per day indefinitely. The courses per day rate increases according to the notional COVAX delivery schedule. Our base case assumption incorporates anticipated scale-up in availability through the COVAX facility with an initial 4000 courses per day in the first quarter rising to 16000, 24000 and 32000 per day in subsequent quarters. We have adjusted the wording in the “Methods: Vaccine programme” subsection to make this more explicit:

> *We assumed courses delivered would increase to 16000, 24000 and 32000 per day in subsequent quarters using the schedule suggested by WHO SAGE (25) modified to reflect the current vaccine landscape (See SI Section 5).*

And later clarified the point about vaccination days to emphasize that this is a model simplification:

> *For simplicity, during the time the vaccine programme is active, vaccination occurs on each day of the week, rather than excluding weekends and holidays.*

We have also amended the sentence in the “Discussion” as follows:

> *Administering vaccine doses in line with projected COVAX availability in a province of roughly 50 million people, it would take around 3 years to reach 60% population coverage.*

To show the impact of scale up compared to a slower rate of roll-out we have added an alternative scenario without scale up where the delivery rate remains at 4000 courses per day over 10 years (see revisions to Table 2). This strategy averts approximately half the deaths compared to a 10 year campaign with scale up, but at substantially lower cost.

We have also added a faster scenario where we assume the entire eligible adult population is vaccinated over 6 months (184K courses per day, or roughly 0.4% of the total population per day, compared to peak doses per day of around 1.5% of the population in the US and UK), finding that this averted roughly twice the deaths of our base case scenario (1 year of vaccination starting with 4000 doses per day and with COVAX-like scheduled increasing rates), but at a substantially higher cost.

*** Imports: It would appear that the model does not take into account imports - "Do not consider external re-introductions" (page 10). How does this affect the results? This could be a discussion point.**

The modelled epidemic behaviour of SARS-CoV-2 in Sindh is governed mainly by natural and vaccine-induced immunity, and not by keeping Rt low through non-pharmaceutical interventions or travel restrictions. Hence the level of imported cases would have to be extremely high to make a change to epidemic dynamics. When incidence is low, transmission is controlled by immunity so imported cases cannot start large outbreaks. Conversely, when incidence is high, there is enough local transmission to sustain the epidemic without requiring imported cases.

To illustrate, we re-ran our base case simulation (and associated non-vaccination scenario) with an importation rate of 10 guaranteed infections/day (not simply exposures) starting coincident with vaccination. The number of averted cases and deaths are both slightly lower (roughly 700K cases with reintroduction vs 900K without, and roughly 9.7K deaths vs 10.1K) 10 years out, with total cases differing by only ~5%.

Imported cases do matter if they introduce new SARS-CoV-2 variants that have different properties from existing variants. We have amended the “Methods” & “Discussion” sections to highlight this. New “Methods: Epidemiological model” text:

> *The modelled loss of protection can represent a range of phenomena, from antibody waning to shifts in the circulating pathogen with time leading to immune escape.*

New “Discussion” text:

> *As demonstrated by recent emergence of novel variants, the underlying epidemiology may shift, as will technological and social trends, including the relative prices of the inputs to the economic estimation. Variants able to escape vaccine-induced immunity may be introduced either through importation or local mutation. This process is partially addressed by considering loss of infection- and vaccine-derived immunity.*

*** Variants: How would the inclusion of variants impact the results? This could be added to the discussion - e.g. the effect of variants is likely to reduce the period of acquired immunity - possibly to less than a year? This would make vaccines more cost-effective if they are protective against variants or less cost-effective if it reduces the vaccine immunity to less than a year. Would it be worthwhile examining vaccine protection of less than a year (worst case scenario for vaccination) to determine what protection duration would no longer be cost-effective.**

We agree that introduction of novel variants is a key concern for projecting vaccine benefits. The difficulty is predicting the time of emergence and specific characteristics of a new variant (e.g. transmissibility, severity, potential to evade immunity). We have attempted to address this using sensitivity analyses on duration of infection- and vaccine-derived protection as well as efficacy. The most pessimistic duration of immunity scenario (1 year for both infection- and vaccine-derived immunity) we considered for the baseline vaccine efficacy (70%) still indicates cost-savings. However, if variants were to emerge at a faster rate than this, we anticipate mismatch between the vaccine and virus because we expect annual vaccine updates (like with seasonal influenza) is the fastest practical outcome, leading to reduced efficacy. For low efficacy scenarios (30%), the resulting median ICERs 10 years out corresponded to order 1000s, indicating very low value.

We have amended the “Discussion” to more explicitly address this issue (we have repeated several sentences from the reply above for context here):

> *As demonstrated by recent emergence of novel variants, the underlying epidemiology may shift, as will technological and social trends, including the relative prices of the inputs to the economic estimation. Variants able to escape vaccine-induced immunity may be introduced either through importation or local mutation. This process is partially addressed by considering loss of infection- and vaccine-derived immunity. For the fastest immunity loss we considered, expected protection durations of a year, a consistently efficacious vaccine (as might be produced by annual updates) can still be cost saving. If variant emergence was more rapid, revaccination with updated formulations might not be able to keep pace, corresponding to lower efficacy. Lower efficacy vaccine (30%) scenarios for rapid protection loss generally resulted in much worse costs per DALY averted (order 1000s of USD per DALY).*

*** One dose versus two-dose vaccine. In the sensitivity analysis in Table 2 it would appear that the adoption of a one dose over a two-dose vaccine changes the number of people vaccinated. But how does it impact vaccine immunity - e.g. what if a person receives only the first dose of a two-dose vaccine, or if there is a delay in receiving the second dose? Is the assumption that the second dose is always provided? If so, this needs to be more clearly stated.**

Our baseline scenario is for a two-dose vaccine, and we set the rate for that assuming the COVAX notional schedule of coverage. We pessimistically assume that only completed courses provide vaccine-derived protection, and have clarified the “Methods: Vaccine programme” subsection text as follows:

Additionally, we assumed 15% of courses would be wasted for reasons such as cold chain failures, incorrect use, or failure to complete second doses (which we pessimistically assume means lack of vaccine protection).

For the Table 2 entry, we intend this to reflect how a one dose vaccine performs within the same delivery constraints (i.e. same number of doses administered means twice as many courses administered), assuming that more vaccines were available.

*** Methods**

o The authors have conducted a very detailed in-depth costing of vaccine delivery which is a major strength of the paper. The analysis would benefit from more clearly disaggregating this costing and presenting it succinctly in Table 1 (or in the results) so that it would be possible to see the total cost per dose administered and the different components that make up this total cost. For example, it would be very interesting to see what proportion of the cost per dose the $3 price of the vaccine contributes towards. Currently, it would appear that the major cost item is this vaccine price, as this changes the results of the analysis from cost saving to no longer cost saving.

Thank you for this suggestion. We have updated Table 1 to include information on how the unit cost was built adding all components together. It is now possible to calculate unit cost per dose using the information presented in the table, and identify cost drivers and their impact.

o Table 1 - what proportion of doses are assumed to be facility-based v. campaign-based? This presumably informs the range for the vaccine cost per dose?

The cost-effectiveness model only considers campaign-based vaccine delivery. We include two types of transport costs, which are mentioned in Table 1: the cost of getting vaccines to the facility and the costs of getting the vaccines from the facility to the campaign sites, both of which need to be considered when understanding total transport costs of campaigns. We presented disaggregated transport costs in Table 1 as felt helpful for others carrying out facility-based costing analysis. We have now clarified the language to ensure these are not mutually exclusive costs.

We had initially calculated human resources costs of facility-based and campaign-based delivery even though only campaign-based delivery was assumed in our model. We had left this cost in Table 1 in case it is helpful for other researchers, but we now feel it confuses the reader so we have taken it out. We will put the information on costs of facility-based human resources in the public domain through other means.

o The authors assume that the vaccine provides protection against infection and disease? How does efficacy play out in the model? Also, the authors use the term "infection-blocking" in the abstract but in the main text describe the vaccine as providing equal protection against infection and disease.

We have clarified this element of the discussion, as well as expanded our scenario analysis to include other efficacy mechanisms.

We use the term “infection-blocking vaccine” to mean that a vaccine prevents someone from getting infected in the first place (and therefore the person cannot get disease either). We distinguish it from (i) a “disease-blocking vaccine” that prevents disease but not infection, and (ii) a “transmission-blocking vaccine” that allows people to be infected but not to infect others. If infection is prevented, then disease cannot occur, so an infection-blocking vaccine is by definition also disease-blocking.

We modified the “Methods: Vaccine programme” subsection text to:

> *For primary vaccine scenarios, we assumed the vaccine is infection-blocking and that protection is complete for some individuals and absent in others (i.e. “all-or-nothing” protection); we considered other vaccine models (“leaky” protection and/or disease-only blocking) as sensitivity studies.*

o Table 1 only shows the base case parameter values but a range is used in the analysis (and presented in Table 2). It would be helpful to the reader to list these all here in Table 1. For example, list base case (70%) efficacy as well as low (30%) and high (90%) etc. This should be followed through to the text - for example, authors state "As alternatives, we considered a higher efficacy (90%) …" But, an efficacy of 30% is also evaluated.
o Organise Table 1 a bit better and maybe include some more of the costs that are mentioned in 'Costs of COVID-19 diagnosis and treatment' section in the table.
o Describe in the methods how the confidence intervals presented in Table 2 are calculated (especially for the ICER).

We have now reorganised Table 1 and added the ranges. We have added a “Methods: Outcome evaluation” subsection:

> ***Outcome Evaluation***
> *For our scenarios, we simulate 100 matched replicates sampling from the epidemiological parameter distribution developed by the fitting process. We calculate the resulting epidemiological and economic outcomes (e.g., cumulative DALYs averted, costs and ICERs at annual increments after start of vaccination) for each intervention scenario matched to the corresponding non-intervention scenario (i.e.,*

by draw from the parameter distribution). We then take the relevant quantiles of these simulation outcomes across the samples.

*** Discussion:**
o How representative is the Sindh province in Pakistan to other LMICs? How generalizable are these results to other LMICs, for example to LMICs which have lower seroprevalence/higher seroprevalence?

We agree this is an important perspective. We have re-ordered the discussion to address this at the outset. Particularly, we have added the following to the "Discussion":

> *The particular context considered, Sindh, is a setting with a young population, high SARS-CoV-2 transmission in the past and limited resources. Many lower- and middle-income settings have similar age distributions and contact patterns, pandemic history, costs, and income levels. As such, we expect these qualitative conclusions to apply broadly, though with detailed quantitative outcomes depending on the location-specific values for those parameters.*

*** Other**
o Two full stops at end of second last paragraph on Page 6
o Define NPI bottom of page 7
o Define DIC bottom of page 9.
o Figure 3 - refers to Figures SY-SZ? Page 13.

Thank you for highlighting these issues; we have corrected them.

Reviewer #2

This was a timely analysis looking at the cost-effectiveness of vaccination in a province in Pakistan. As COVAX begins to distribute vaccines, it's helpful to understand the expected impact and cost-effectiveness of this vaccine distribution. The authors do an excellent job looking at all different types of

Methods

1. "Leaky protection" - please describe what this means in comparison to non-leaky protection- and the rationale for choosing this approach.

We clarified the definition of this assumption, and also expanded our analysis to consider alternative model mechanisms. We have modified the text in "Methods: Vaccine programme" subsection to:

For primary vaccine scenarios, we assumed the vaccine is infection-blocking and that protection is complete for some individuals and absent in others (i.e. “all-or-nothing” protection); we considered other vaccine models (every exposure tests the efficacy independently, i.e. “leaky” protection, and/or disease-only blocking) as sensitivity studies.

2. "Given the emphasis on prioritising older adults in WHO's vaccine prioritisation roadmap (27), we considered two scenarios for distribution: either individuals 15+ years old for the duration or individuals 65+ years old for the first quarter before shifting to 15+. For all scenarios, we assume vaccine doses are uniformly (i.e., proportional to fraction of population) distributed in the targeted populations."

I recommend considering a third scenario- which may be particularly in resource limited settings- the impact and cost-effectiveness of targeting those 65+ for the entire duration of the time period.

Because the population of Sindh is very young (similar to many LMIC settings), the proportion of 65+ year olds is relatively small (roughly 4.5%). We have adjusted the lowest courses per day scenario (initially 4000) to continue for 6 months before switching, but for the other scenarios extending the focus on the 65+ population would result in multiple courses per individual. This change to add an extra quarter of prioritization of 65+ individuals increases deaths averted, but not substantially.

3. "For COVID-19 deaths we estimated age-specific DALYs using the premature-death method by Briggs (29,30) which builds on standard life-table methods to estimate the discounted years of life lost adjusting for age-related quality-of-life (QoL) in the general population, and also allows for inclusion of different baseline morbidity and mortality assumptions."

Traditional DALY calculations would be difficult in COVID, particularly in LMICs where the average age of COVID death is probably greater than the average life expectancy. I had to read the Briggs paper in depth to be able to understand the approach- I think it's worth a few-sentence summary here and address how this specifically is handled in your application of the Briggs approach.

We have modified the text to read:

For COVID-19 deaths we estimated DALYs, guided by the approach presented by Briggs et al. (29). We generated age at death in 5-year age-bands, and then applied age-specific life-expectancy at death using national life-tables for Pakistan (United Nations estimates for 2015-2020 (30)). We adjusted Years of Life Lost (YLLs) considering the overall level of disability for any remaining years of life using data on QoL by age-band from Zimbabwe (31) since all other countries with available data were high-income. However, in our base case analysis, we did not adjust standard life-tables to take into account any reduced life-expectancy due to specific comorbidities associated with COVID-19. As a sensitivity analysis, since risk of severe COVID-19 is higher for people with comorbidities (32), we modelled an alternative scenario in which half of COVID-19 related deaths were assumed to occur in individuals with higher baseline mortality (Standardised Mortality Ratio = 1.5) and 10% lower baseline QoL. We calculated the average DALYs per death using both 3% (base case) and 0% discounting (SI Table S7).

4. Scenario analysis: There were many scenarios evaluated, which were helpful. However, what if there is differential immunity for vaccine and natural immunity- like we see with influenza? E.g. what if the duration of vaccine immunity is 2.5 years and duration of natural infection immunity is 10 years? To what degree does this impact cost-effectiveness?

Increasing the duration of natural immunity can increase (if the marginal contribution of vaccination pushes population over the herd immunity threshold) or decrease the benefits of vaccination (if effort vastly exceeds the herd immunity threshold). We did not consider a 10 year duration of infection-derived protection specifically, but for life-long infection-derived protection (and other parameters consistent with our baseline scenario), the cost per DALY averted becomes 1088 (-57K to 69K to larger uncertainty due to potential extinction), compared to the baseline result of 27.9 (1.7-40.9).

5. Distribution assumption: The fact that this model examines the COVAX distribution is very useful, particular for the early phase of vaccination. However, once high-income countries feel satisfied with their vaccination rates (ugh. so much for vaccine equity.), there may be more vaccine available for LMICs (or could be advocated for). How much more cost-effective would the vaccination campaigns be if you could double/triple/quadruple the number of vaccines per day (say, starting in mid 2022?)? If this is shown to be vastly (or even marginally) more cost-effective, the findings of this manuscript could be used to further advocate for more vaccines through COVAX or other distribution mechanisms.
I could also imagine a scenario in which you have a massive vaccination campaign every so-many-years (depending on the duration of vaccine-induced immunity)- and that could be substantially more cost-effective than a slow drip of immunization. (So, what if we could vaccinate most of the 65+ population every 5 years or so?) Any of

these different distribution scenarios could have the power to change and advocate for vaccines for LMICs and I strongly suggest the authors pursue these scenarios.

From another reviewer’s comments we realise it may have been unclear that our base case assumption incorporates anticipated scale-up in availability through the COVAX facility with an initial 4000 courses per day in the first quarter rising to 16000, 24000 and 32000 per day in subsequent quarters. We have adjusted the wording in the “Methods: Vaccine programme” subsection to make this more explicit.

Reiterating our previous reply about considering expanded dose-rate scenarios: to show the impact of scale up compared to a slower rate of roll-out we have added an alternative scenario without scale up where the delivery rate remains at 4000 courses per day over 10 years (see revisions to Table 2). This strategy averts approximately half the deaths compared to a 10 year campaign with scale up, but at substantially lower cost.

We have also added a faster scenario where we assume the entire eligible adult population is vaccinated over 6 months (184K courses per day, or roughly 0.4% of the total population per day, compared to peak doses per day of around 1.5% of the population in the US and UK), finding that this averted roughly twice the deaths of our base case scenario (1 year of vaccination starting with 4000 doses per day and with COVAX-like scheduled increasing rates), but at a substantially higher cost.

Minor comments

1. "For the non-fatal outcomes, and in the absence of specific DALY data, we used Quality Adjusted Life Years (QALYs) reported by Sandmann et al. (28) based on pandemic influenza studies treated one QALY as equivalent to one DALY averted."
I suggest adding the word 'gained': "… treated one QALY gained as equivalent to one DALY averted."

Thank you, we have incorporated this change.

Reviewer #3

This study estimated COVID-19 cases and death over 10 years under various vaccine scenarios in a population of 48 million people for a Pakistani province. The simulation model is a previously published compartmental transmission model under an extended SEIRS+V structure including birth, death, and age-strata. The model is calibrated to new daily cases and deaths in the Sindh province from April to September 2020 and validated until January 2021. The authors conducted a cost-effectiveness analysis of various vaccine scenarios compared to no vaccination over 10 years. Sensitivity analyses include the length of vaccination campaign, cost per dose, natural immunity loss and duration of vaccine protection, etc. The study concluded that COVID-19 vaccination is likely to be cost-effective

if the cost is low and vaccine has good protection against infection in low- and middle-income countries.

This study is well done. Long-term model projections under various natural immunity loss and vaccine protection waning scenarios are particularly insightful. I have the following comments to the authors.

Major comments

1. Contact patterns changes were estimated using Google Community Mobility indicators and school closures were considered using government response tracker. The authors assumed there is no further social distancing measures after May 2021. This is a very strong assumption. Have the authors considered face mask use data in Sindh and its effect on transmissibility? Given recent government interventions responding to third waves of infection in Europe and face mask use recommendations in many countries, I recommend the authors at least adding a discussion on continuous use of non-pharmaceutical interventions and prolonged changes in contact patterns after 2021.

We agree that long term patterns of non-pharmaceutical interventions could impact health outcomes, and therefore economic benefit of vaccination programmes. However, what will happen with these, and how to appropriately cost their implementation and impact, is extremely uncertain. We have added this explicitly to the "Discussion" as a limitation of these projections:

> *We do not consider future non-pharmaceutical interventions beyond May 2021 or innovative coordination with vaccination. If there are substantial changes from the impacts integrated into the fitted estimates of local transmission, our projections will not reflect those.*

We have not directly considered mask usage data, but any impact to-date is implicitly incorporated in the fitting process, and thus would be included in projections (via lower transmissibility multiplier). We do not explicitly contemplate future additional gains to masking behaviour (e.g., improved technology, compliance, or uptake).

2. There are several optimistic assumptions about vaccine. First, 10% wastage was assumed. I would like to see if there is any evidence backing up this assumption which seems to be very low.

We had initially assumed a 10% wastage rate, deemed to be a plausible average of the wastage of one-dose vials (~5%), the most typical presentation of Covid vaccine candidates whose details were available at the time the model was set up, and the wastage experienced during campaigns (~15%), as the cost model covers a mix of fixed sites vaccines delivery and vaccination campaigns. This has now been increased to 15% to reflect that the vaccines currently available through COVAX come in multi-dose vial presentations. Increasing the

wastage does decrease expected courses per day, but we had already pessimistically rounded down to the nearest 1000 per day (4000), which is unchanged by increased wastage.

Second, the authors assumed that an effective vaccine provides full protection against infection. I would recommend an additional scenario analysis that relaxes the full protection assumption or a more in-depth discussion on this point. For example, if the vaccine is disease-modifying only, or gives partial protection against infection and reduces transmissibility.

Thank you for this suggestion. We have now also produced projections with other efficacy mechanism assumptions. We quote some specific ones in Table 2, including disease-blocking and leaky vaccines. In general, disease-only blocking vaccines are not as effective (as expected) but relative performance along other dimensions (e.g. initial target population, efficacy, protection duration) remains qualitatively similar.

3. One page 6, could the authors clarify this vaccination prioritization strategy, "individuals 65+ years old for the first quarter before shifting to 15+"? Does this mean 65+ only in the first quarter of the first year of vaccination, or prioritized annually like a seasonal flu vaccination scenario?

The former; we have now modified that phrase to read “individuals 65+ years old for the first two quarters of the first year before shifting to 15+”. This change also includes a change that 65+ targeting lasts for two quarters not one quarter.

4. In the base case analysis, future cost is discounted at 3% annually, and health outcomes are discounted at either 0% (base case) or 3%. Equal rate of discounting is the more common practice, though there are some debates about this practice (https://www.ncbi.nlm.nih.gov/pmc/articles/PMC5999124/). Was there a particular reason that the authors decided to not discount health outcomes in the base case?

We originally used 0% discounting of health outcomes in the base case because of WHO guidelines in this area [https://apps.who.int/iris/bitstream/handle/10665/329389/WHO-IVB-19.10-eng.pdf]. However, we agree with the reviewer’s point that WHO’s approach is not widely accepted in the health economics community, and hence now switch to 3% in the base case, and 0% in sensitivity analysis.

5. The author mentioned in the Discussion that 4000 doses/day would need to be continued for a long time to have a large impact. It would be more informative to give readers some ideas on the vaccine coverage over time.

We agree that this perspective is useful. We have modified the “Discussion” to include the following:

Administering vaccine doses in line with projected COVAX availability in a province of roughly 50 million people, it would take around 3 years to reach 60% population coverage.

Reviewer #4

This is an interesting, well-conducted, and timely analysis on the impact of targeted vs general COVID vaccination strategies in low and middle-income countries.

My major comment is that the authors evaluated only two scenarios of vaccine distribution: 1) >65 first followed by the entire adult population, and 2) all persons 15+. Given the complex tiers of vaccine rollout in high-income settings (stratified by healthcare worker status, age, age + comorbidities, essential workers, etc) it would be useful to project the impact of other distribution strategies. For example, the authors find that vaccination of individuals >65 prevents slightly more deaths than mass vaccination, which is not surprising given the smaller proportion of older individuals in low-income settings. However, would vaccinating individuals >50 provide greater benefits? What would be the added benefit of a tiered approach with respect to age and co-morbidities? Projections of vaccine benefit by population heterogeneity (particularly co-morbidities) may be difficult due to the simpler compartmental model design, but it would be helpful to discuss the potential impacts of these strategies or their benefit over a mass vaccination approach in the discussion section.

While we agree that these prioritization strategies are in principle beneficial in high-income settings, in practice the data and health systems to support them are not typically available in LMIC, nor is the detailed data necessary to model them explicitly (e.g. how these individuals mix with others in the population).

To investigate this question, we looked at the impact that initially prioritising 50+ year olds instead of 65+ year olds would have. Given the particular demographics of Sindh and our quarter-based transition of age-based prioritization, we find that changing to 50+ would not be expected to change the transition timing, so would represent a minor tradeoff in transmission- versus disease- prevention. Indeed, when simulating prioritizing from 50+ initially, we find slightly fewer deaths prevented (9.1K median deaths averted vs 10.1K in the 65+ prioritization) and slightly more cases averted (1M median cases averted vs .9M). This does not capture the potential benefits of a more continuous prioritization scheme (e.g., starting at higher ages and lower distribution age at a finer resolution) such as has been used in high-income settings, but any benefits of such an approach rely on the assumption that the more complicated deployment scheme will not cost more (or decrease/delay vaccine uptake, etc). That assumption may not reliably apply in lower- and middle-income settings. Given the small differences and potential

cost, logistic and rollout implications which we are unable to properly parameterise, we have opted not to include this scenario in the manuscript.

It would be helpful for the authors to address the impact of vaccine hesitancy and the potential correlation between risk-taking behavior and refusal to uptake vaccination. In particular, vaccinations have a complex political history (ie with polio eradication efforts) and targeted violence toward vaccination workers.
https://www.npr.org/sections/goatsandsoda/2021/02/24/968730972/pakistans-polio-playbook-has-lessons-for-its-covid-19-vaccine-rollout

https://gallup.com.pk/wp/wp-content/uploads/2021/01/Gallup-Covid-Opinion-Tracker-Wave-9-pdf.pdf

Given a high distrust of COVID-19 vaccination, would a country such as Pakistan avert greater illness using mass vaccination for all age groups? Perhaps the impact of vaccine refusal rates can be explored in a sensitivity analysis. Perhaps this can also be mentioned in the discussion section.

In principle, our model framework already incorporates vaccine hesitancy, because we look at different scenarios about vaccine uptake that incorporate both supply (availability of vaccines) and demand (desire for people to be vaccinated) factors. We are not however able to look at associations between receiving vaccination and other risk-taking behaviour which may lead to lower or higher risk of transmission. We have added this as a limitation in the “Discussion”

> *We also assumed that within a particular age group, there is no association between probability of getting vaccinated and risk of disease. This may not be accurate if for example vaccination targets people with comorbidities (and hence higher risk of severe COVID-19 disease), or people who are risk averse (and hence less likely to be infected) are also more likely to get vaccinated.*

The authors assume an exponential distribution for waning immunity—this would imply a fast waning early on. It is possible that immunity declines more slowly at first. What are the implications of choosing this distribution?

In the short term, an alternative waning immunity assumption (e.g. a logistic function with a steep slope) will increase vaccine impact in the earlier time period after vaccination starts. Longer term, the model is simply vaccinating individuals in eligible states, and if the average time in vaccinated state is comparable (i.e., the suggested slow initial waning is balanced by more rapid later decline), then the average amount of immunity in the population will equilibrate at a comparable level.

We do not have any good evidence that immunity waning has a particular time distribution. However, rapid declines in antibody titres following natural immunity (e.g. Ward et al. https://doi.org/10.1016/j.lanepe.2021.100098) or one dose of AstraZeneca vaccine (Voysey et al. https://doi.org/10.1016/S0140-6736(21)00432-3), and reports of reinfection of recovered patients months after the initial infection (e.g. Hansen et al. https://doi.org/10.1016/S0140-6736(21)00575-4), suggest that at least some individuals lose immunity rapidly.

Introduction:
The authors state: "For all vaccine scenarios, we assumed the vaccine provides protection against infection (not just disease) and that protection is tested with each exposure in the model (i.e. "leaky" protection).

It would be helpful to define the concept of leaky vaccine more clearly for a lay audience of policymakers and researchers.

Thank you for this suggestion. We have clarified the definition per the following; additionally, we now consider more vaccine mechanism scenarios:

> *For primary vaccine scenarios, we assumed the vaccine is infection-blocking and that protection is complete for some individuals and absent in others (i.e. “all-or-nothing” protection); we considered other vaccine models (every exposure tests the efficacy independently, i.e. “leaky” protection, and/or disease-only blocking) as sensitivity studies.*

Do the authors have data on the age distribution of comorbidities associated with COVID-19 severity in Pakistan? If so, how were these data incorporated into the model?

Because the increased risk of severe disease attributable to particular comorbidities is unknown, particularly in settings such as Pakistan, we did not explicitly incorporate comorbidities into the base case model. Instead, we used age-dependent infection-hospitalisation and infection-fatality ratios based on international data. Although not based on empirical data, as a sensitivity analysis we modelled an alternative scenario which assumed that half of Covid related deaths were assumed to occur in individuals with higher baseline mortality (standardised mortality ratio of 1.5) and lower (10% reduction) baseline Quality of Life. Results of this sensitivity analysis are included in Figure 5 and Table 2.

The population mixing assumptions would likely have a strong impact on the results, particularly for herd immunity. Can the matrix assumptions be varied? If not, what are the likely implications of misspecifying the matrix, in terms of choice of vaccination strategy?

We did not consider varying the mixing matrices. There would be counterbalancing effects to doing so (e.g., different conclusions from the fitting). Perhaps more critically, however, this class of model is generally not capable of capturing effects like household isolation (i.e. household contact patterns remain and can reach across the entire population, rather than saturating a micropatch in the population). These misspecification issues are addressed somewhat by fitting to get the best approximation given the available mechanisms. However, these misspecifications are mostly problems for addressing detailed non-pharmaceutical interventions (and the interaction of vaccine programmes with NPIs). That question is out of scope for this work as it would demand a far more detailed model to address and data to appropriately parametrize the necessary elements within a model is limited for LMIC settings.

We now discuss these issues as part of the model limitations in the Abstract:

> *These projections are limited by the mechanisms present in the model. Because the model is a single-population compartmental model, detailed impacts of non-pharmaceutical interventions (NPIs) such as household isolation cannot be practically represented or evaluated in combination with vaccine programmes. Similarly, the model cannot consider prioritizing groups like healthcare or other essential workers. Additionally, because the future impact and implementation cost of NPIs is uncertain, how these would interact with vaccination remains an open question.*

As well as the Discussion:

> *We do not consider detailed non-pharmaceutical interventions or innovative coordination with vaccination. If there are substantial changes from the impacts integrated into the fitted estimates of local transmission, our projections will not reflect those.*

For Table 1, it would be useful to list the range of values used in the sensitivity analyses in addition to the base case values.

We agree and have revised Table 1 to include these values.

Much of the discussion section provides a summary of the findings. It would be useful to also contextualize the finding in terms of the strengths and limitations of the model and inputs used. How generalizable are the findings to other countries and what are the factors that most impact generalizability? Eg age structure or mixing in the population?

We agree generalizability is an important consideration. In addition to the changes highlighted in our previous response about model limitations, we have also added this text to the discussion:

> *The particular context considered, Sindh, is a setting with a young population, high previous SARS-CoV-2 transmission and limited resources. Many lower- and middle-income settings have similar age distributions and contact patterns, pandemic*

history, costs, and income levels. As such, we expect these qualitative benefits to apply broadly, though with detailed quantitative outcomes depending on the location-specific values for those parameters.

Reviewer #5

This study aims to assess the health impact, economic impact, and cost-effectiveness of COVID-19 vaccination in Sindh province, Pakistan, using a combined epidemiological and economic model.

Comments

The authors apply a previously published compartmental model, providing the relevant citation and a concise summary in brief here.

A technically appropriate methodology of Bayesian inference via Markov Chain Monte Carlo has been used to fit elements of the model, and the authors have undertaken a thorough set of out-of-sample validations.

"Vaccine doses are distributed amongst individuals in the Susceptible and Recovered compartments; Susceptible individuals become Vaccinated, while Recovered are unchanged."
Can the authors please explore and discuss whether, by leaving Recovered unchanged, it is realistic to assume that the Recovered population is the same as the Recovered and Vaccinated population within the model?

We have adjusted our model to include an Recovered-and-vaccinated (R_V) compartment. This compartment assumes the completely immunizing protection associated with the Recovered compartment, but allows for waning into the Vaccinated compartment and thus potentially additional duration of protection. This did change quantitative results, but was a marginal contribution compared to other factors - e.g. almost all of the roughly 3000 additional deaths prevented in the base scenario are due to extending prioritization amongst 65+ by an additional quarter. The change to add an R_V compartment only contributes order 10s of deaths difference.

The authors appropriately acknowledge the limitation of the current model by not including the possible impact of variants.
"As demonstrated by recent emergence of novel variants, the underlying epidemiology may shift, as will technological and social trends, including the relative prices of the inputs to the economic estimation. Given that core uncertainty, the intervals ought to be thought of as on our estimate of the central trend, rather than as reflecting the volatility in the system."

The authors have suitably provided the CHEERS checklist.
