# Supplementary material for: COVID-19 vaccination in Sindh Province, Pakistan: A modelling study of health impact and cost-effectiveness
Source: PLoS Med. 2021 Oct 4;18(10):e1003815. doi: 10.1371/journal.pmed.1003815 (PMC8523052; doi:10.1371/journal.pmed.1003815)
Supplement: Attachment — Submitted filename: ResponsetoReviewers2.pdf [file pmed.1003815.s004.pdf]

Please see below for our responses to items from the reviewers. Our responses are in blue. Where we are including revised text from the manuscript, we have indented and italicized that text.

Review #1:

Minor Essential Revisions:

Happy with the responses and revisions that the authors have made to their manuscript. All revisions add value and necessary clarity.

Methods:

The authors have made clarifying adjustments to their Table 1. I would suggest the following additional adjustments:

- Include a vaccine price of $6 in price range as this is used in Figure 4.
- Regarding vaccine wastage, Page 7 of the SI should read "times 85% (for 15% wastage)", not 90%. Also, should this not be updated in Table 1? Wastage is 15% of vaccine procurement price per dose (not 10%)?
- Duration of vaccine-induced immunity. In the results look at 1, 2.5., 5 and life-long. In Table 1, don't mention 5 year.
- The authors have added a lot more information regarding the costing to Table 1. Thank you, it is clearer. However, I think it would still be worthwhile having sub-totals showing the cost of delivery per dose that is mentioned on page 14 as $1.01 as a quick calculation that I did I could not replicate this figure. In the SI, it states that the cost of an AD syringe and safety box is also included. Is this included in the $3 or not. It might account for my inability to replicate the cost.

In response to these items:

- We have added a $6 vaccine to table 1.
- We have corrected this in the SI. This was an oversight in our previous update to the SI from 10% to 15% vaccine wastage. Wastage of 10% is assumed for other immunisation supplies
- We considered 5 years as part of our full set of scenarios, but did not select that as one of our focal scenarios. The explicit quantitative results are available in the full data set from https://doi.org/10.5281/zenodo.5070957, which we have added to the data availability statement.
- We have updated Table 1 to include the cost of immunization supplies, as well as clarified the discussion in the Supplementary Information:

  We assumed a procurement price of the vaccine of $3 per dose (10), with an additional 10% added for freight costs. We also account for the costs of an AD syringe and safety box. We assumed a 15% wastage for vaccines, the wastage in line with published data from immunisation typically experienced during campaigns, and 10% wastage for immunisation supplies (11).

Results

Thank you for adding the additional scenarios – namely, slow and fast roll-out. I am surprised that less deaths are averted in the Fast roll-out scenario relative to the Slow roll-out. This would suggest that there are benefits to a slow roll-out – cheaper and averts more deaths? Surely there are more benefits to vaccinating the population quickly in a short space of time? Or is this mitigated by the vaccine-induced immunity of 2.5 years. I am also surprised that the 1 dose regimen is cost-saving. Is this because it is essentially half the price per vaccine (e.g. $1.5)? That does not really make sense.

The faster rollout scenario indeed rapidly increases population-level immunity. But as the reviewer notes, we have assumed transient immunity, and only considered a one-time fast-rollout scenario, not ongoing coverage at the level (unlike our reference scenario). Eventually, that protection fades and the pandemic returns. We did not consider factors like a rapid global campaign eliminating SARS-CoV-2.

The 1 dose regimen, for the baseline efficacy, is indeed cost-saving. The reviewer's example is approximately the correct intuition, though for the $3 per dose scenario, the 1 dose regimen is $3 (plus assorted other costs) while the two dose vaccines are $6 per regimen (again, plus assorted other costs).

Reviewer #2:

The authors have substantially improved the manuscript in response to reviewer comments. However, upon re-read of the updated version, in light of everything we know about vaccination at this moment in time I have one major concern:

Who is asking whether or not vaccination is cost-effective (and is basing vaccine delivery strategies based on cost-effectiveness)? Across all LMICs that I've worked with throughout the pandemic, the cost of the vaccine does not seem to be the limiting factor in all of this (particularly as the cost/dose is not the $10 that the authors cite as a worst-case scenario) [https://www.who.int/docs/default-source/coronaviruse/act-accelerator/covax/costs-of-covid-19-vaccine-delivery-in-92amc_08.02.21.pdf]. The costs are low, the effect is clearly high. The real issue remains access to vaccines in the first place (while high income countries eat up the world supply) and within country distribution.

I do see some marginal utility in this analysis with regard to type of scale-up strategy (65+ versus everyone)- and the analyses were all very elegant… but ultimately, by the time more guidance is needed, particularly with the introduction of variants that may reduce the response to current vaccines (as the authors have added into the discussion)- the question on policy maker's minds may then rather (and rightly) become the cost-effectiveness of booster vaccines for some proportion of the population instead of just focusing on initial vaccines.

The authors should do a more robust job convincing the reader why this one regional analysis is important, and what it adds to the literature - or adapt the model explicitly to look to the question of variants and boosters.

This work was conducted in response to a direct request from the Ministry of National Health Service Regulation and Co-ordination in Pakistan in collaboration with the Government of Sindh. We have also had similar requests from five other low- and middle- income country governments, covering substantial populations in Africa and Asia, and we are aware of similar requests to groups like ours around the globe. We agree with the reviewer that these requests are motivated by supply considerations, like the availability from COVAX. These supply issues, combined with the extent of the public health crisis, are leading countries to evaluate direct purchase of COVID-19 vaccines from a range of suppliers, with varying price and performance characteristics.

We respectfully disagree that the "price is low" in all settings. Pakistan currently has USD 8 in total per capita within the public health sector to spend on primary health care. High coverage with a COVID-19 vaccine would represent a substantial portion of the health sector budget, squeezing other essential health services. To avoid that outcome, these costs would ideally be met by special funding outside the usual health sector budget; however, in order for the case for this to be made, the country needs information on the budget impact and economic value of the intervention.

We agree with the reviewer that there is a clear and obvious need for rapid COVID-19 vaccination in LMICs, but the unfortunate financial reality in many LMICs means that economic analysis of even this most urgent public health intervention is important to inform LMIC government decisions.

As the reviewer suggests, we do find that a vaccination programme at a lower price of $3/dose (close to the cost of vaccines purchased under the COVAX Committed Purchase Agreement) is very likely to be cost-effective. However, Pakistan is also thinking of purchasing additional vaccine doses on the open market due to the limited supplies available through COVAX. Under these arrangements, the prices of vaccine doses are likely to be closer to our upper assumption of $10/dose.

The reviewer correctly asks why one regional analysis is important. Scientifically, it is important as it is the only analysis that we are aware of at this time that demonstrates the cost-effectiveness of vaccines in LMICs, and provides an ICER estimate. For example, this was the only study on cost-effectiveness of vaccines presented to WHO SAGE to inform their vaccine policy. The WHO SAGE COVID-19 working group has called for more epidemiological and economic modelling studies on COVID-19 vaccines in LMICs. While cost-effectiveness is always a context specific assessment, this study demonstrates that vaccination should still be considered even in a generally younger population with a very limited health budget.

However, in the absence of global or multi-country analyses, an analysis set in a specific location provides vital evidence both for the subject country, as well as to highlight similarities and differences with studies set in high-income countries that currently make up almost all the published literature.

Many of the elements of this response are already present in the manuscript, but we have further emphasized the summary motivation of this work by adding the following to the introduction:

Given that this supply may not be sufficient, countries may need to purchase additional vaccines, and to do so need to be able to quantify the costs and benefits of various vaccination programmes to compare to other health sector investments.

Minor revisions:

Methods: Suggest changing "highly developed settings" to "high income countries"

We have made this alteration.

Reviewer #3

The authors have addressed all my comments from the last round.

Reviewer #4

The authors have sufficiently addressed the reviewer comments in this revised manuscript. I have a few additional suggestions.

Abstract:

"Varying these assumptions, we generally find that prioritizing the older (65+) population prevents more deaths, but broad distribution from the outset is economically comparable in many scenarios, and either scheme can be cost-effective for low per-dose costs."

This sentence is a bit long and hard to follow.

We have revised this sentence to:

Under a broad range of alternative scenarios, we find that initially prioritizing the older (65+) population generally prevents more deaths. However, unprioritized distribution has almost the same cost-effectiveness when considering all outcomes, and both prioritized and unprioritized programmes can be cost-effective for low per-dose costs.

"However, high vaccine prices ($10/dose) may not be cost-effective."

This statement seems very tentative. Can it be made more definitive? For example: We found vaccination was not cost-effective at high vaccine costs (10/dose).

This statement is qualified because of the uncertainty in what will be. For some of the scenarios that we considered (and for some thresholds of cost-effectiveness), that price means the vaccine programme would not be cost effective; for other circumstances, it would. We have clarified the qualifier by changing the sentence to:

High vaccine prices ($10/dose), however, may not be cost-effective, depending on the specifics of vaccine performance, distribution programme, and future pandemic trends.

"These projections are limited by the mechanisms present in the model."
This sentence is a bit vague and can be removed. The following sentence could start with: Limitations of this study include…

We have revised this paragraph a bit more broadly, to have the first sentence succinctly enumerate the broad categories of limitations and then flow into more detailed explanation. It now reads:

This study is limited by model approximations, available data, and future uncertainty. Because the model is a single-population compartmental model, detailed impacts of non-pharmaceutical interventions (NPIs) such as household isolation cannot be practically represented or evaluated in combination with vaccine programmes. Similarly, the model cannot consider prioritizing groups like healthcare or other essential workers. The model is only fitted to the reported case and death data, which are incomplete and not disaggregated by, for example, age. Finally, because the future impact and implementation cost of NPIs is uncertain, how these would interact with vaccination remains an open question.

"Preventing severe disease is an important contributor to this impact, but the advantage of focusing initially on older, high-risk populations may be smaller in generally younger populations where many people have already been infected, typical of many low- and -middle income countries, as long as vaccination gives good protection against infection as well as disease."

This is a very long sentence. It would be helpful to divide it into a few shorter sentences and clarify the conclusion in more certain terms.

We agree and have revised the paragraph to:

COVID-19 vaccination can have a considerable health impact, and is likely to be cost-effective if more optimistic vaccine scenarios apply. Preventing severe disease is an important contributor to this impact. However, the advantage of prioritizing older, high-risk populations is smaller in generally younger populations. This reduction is especially true in populations with more past transmission, and if the vaccine is likely to further impede transmission rather than just disease. Those conditions are typical of many low- and -middle income countries.

"What Do These Findings Mean?"

This section can be modified to incorporate the implications of the study on COVID vaccination policy.

It might be worth mentioning in the discussion section that other age stratified vaccination scenarios were not modeled because of the lack of epidemiologic data and the difficulty of

conducting stratified vaccination for most health systems. Otherwise it may be somewhat intitutive that the health impact of prioritizing persons >65 years in LMICs is low because of the small number of people in that category.

We have modified the Findings section to include a new bullet:

> *The results suggest that low- and middle-income countries (LMICs) see less benefit to initially prioritizing vaccination of older (65+) populations compared to unprioritized distribution. Factors outside this analysis, like cost differences between prioritized and unprioritized programmes, will further influence the preferred approach.*

We have also added the following to the Discussion regarding the prioritization schemes we considered:

> *We did not consider more complex age-prioritization strategies, such as vaccinating 5-year age bands until reaching a particular coverage and then prioritizing the next lower band, because such a programme would entail more complex administration costs and need a model validated on age-specific past cases. We do not have the appropriate data to support such an analysis.*

Methods, costs

"Following WHO guidelines, we used a 3% discount rate for future costs and for annualising capital investments, while health outcomes are discounted at either 0% (base case) or 3% (35)."

Most economic guidelines recommend using the same discount rate for health benefits and costs. It would be useful for the authors to provide an explanation behind the rationale to not discount health benefits (but only discount costs) in their sensitivity analysis. It would be useful to provide a sensitivity analysis where neither costs nor health benefits are discounted.

We have corrected the above sentence so that it now accurately reflects the use of 3% discounting for both costs and DALYs, and cites WHO guidelines to justify the discount rate in out base case analysis:

> *"Following WHO guidelines, we used a 3% discount rate for future costs and for annualising capital investments, while health outcomes are discounted at either 3% (base case) or 0% (35)."*

Table 1

It would be helpful for the authors to conduct more sensitivity analyses as many of the parameters are highly uncertain, particularly COVID natural history, vaccination

efficacy/duration. These could be presented in the supplemental appendix and referenced in the text. Costs could also be varied more widely. Vaccination is cost-effective at $3 per dose but not cost effective at $10 per dose—this is a wide range. The ICER for vaccination at $10 per dose is very high. At what approximate threshold does the vaccine no longer become cost effective? Similarly, a 30% vaccine efficacy is unlikely but what is the impact of a 50% efficacious vaccine?

We have provided substantial sensitivity analyses in the appendices addressing most of these issues. To address the issue of the wide range price, we have now added an intermediate ($6) vaccine price scenario to table 1. However, we have not been able to conduct price threshold analyses. While threshold analyses are useful, that kind of calculation in our context is difficult to interpret because of the lack of an explicit cost-effectiveness threshold in Pakistan, high level of uncertainty around many parameters, and large number of scenarios we need to consider.

However, we have made the necessary simulation results for anyone wishing to conduct such an analysis at https://doi.org/10.5281/zenodo.5070957; this has also been noted in data availability.

Table 2:

It may be helpful to replace "dom" with "cs" for cost-saving as dom usually refers to dominated (a strategy that is more costly and less cost-effective).

We have replaced “dom” with “cs”.

Discussion:

"Many lower- and middle-income settings have similar age distributions and contact patterns, pandemic history, costs, and income levels. As such, we expect these qualitative conclusions to apply broadly, though with detailed quantitative outcomes depending on the location-specific values for those parameters."

Could the authors be more specific about which countries these results would be generalizable to? Other countries in South Asia, or Asia? Or Asia and Africa?

We do not wish to imply generalisability for other specific settings, given the differences in epidemiology, costs and cost-effectiveness thresholds. But we take the point, and believe our findings are a good starting point for understanding cost-effectiveness in LMICs more generally, and particularly that should be expected to be distinct from HIC conclusion. Thus, we have revised the first sentence to highlight when our findings may apply:

> *The age distributions, contact patterns, pandemic history, costs, and income levels of most low- and middle-income countries are likely more similar to those used in this analysis than to those in comparable studies for high income countries. While the*

specific quantities found in this analysis are unlikely to apply explicitly, the qualitative differences from high income countries likely will.

"Under our base case assumptions, a single year of vaccination would cost an additional USD 2 million compared to no vaccination, and would avoid 70,000 DALYs resulting in an ICER of USD 28 per DALY averted."

Could these results be specified in cases and deaths averted since DALYs are harder to understand intuitively?

We agree that cases and deaths averted are easier to understand intuitively, and hence we do report these figures in the results tables. However, DALYs averted are recommended in guidelines on standardised cost-effectiveness analysis (which allows for comparability across settings and interventions). Moreover, the main health outcome in our paper reflects both mortality and morbidity. We would argue that this is the most comprehensive measure that captures differences in severity of cases (e.g. mild cases vs deaths) and the remaining life years lost by deaths in people of different ages (Briggs and Vassall, 2021), and should be the metric used to both assess the health burden and impact of interventions for COVID-19..

"This model also indicates that these benefits are not particularly dependent on the target population."

I don't think this can be concluded without conducting an age stratified analysis and including healthcare workers, which is not done in this present study. It is unlikely that vaccinating the general population would result in higher benefits than vaccinating healthcare workers.

We agree, and have adjusted the wording here to clarify that we are only concluding that relative to age-based prioritization. The adjusted text reads:

This model also indicates that these benefits are not particularly dependent on the age group targeted.
