# Supplementary material for: COVID-19 vaccination in Sindh Province, Pakistan: A modelling study of health impact and cost-effectiveness
Source: PLoS Med. 2021 Oct 4;18(10):e1003815. doi: 10.1371/journal.pmed.1003815 (PMC8523052; doi:10.1371/journal.pmed.1003815)
Supplement: Attachment — Submitted filename: Sindh Response Letter, part III.pdf [file pmed.1003815.s005.pdf]

Response to reviewer and editor comments:

Reviewers:

Reviewer 1 requested that we update Table 1 to reflect changes elsewhere in the manuscript: “The authors have addressed all my comments from the last round. I however feel that the authors still need to adjust table 1 to reflect the "15% wastage for vaccines, and the 10% wastage for immunization supplies" as indicated in the text.”

We have made this modification. The table entry now reads: “15% of vaccine procurement price per dose, 10% of immunization supplies procurement price per dose”.

There were no other reviewer comments to address.

Editorial comments:

1. Please format your references as follows:
a. Please use the PLOS Medicine style reference call outs throughout the text, noting the absence of spaces within the square brackets, e.g., "... every country) [1,2]." https://journals.plos.org/plosmedicine/s/submission-guidelines#loc-references.
b. Please ensure that journal name abbreviations match those found in the National Center for Biotechnology Information (NCBI) databases, and are appropriately formatted and capitalized. https://journals.plos.org/plosmedicine/s/submission-guidelines#loc-references. Please ensure that weblinks are current and accessible to date.

We have switched to square brackets for citations. We have updated journal abbreviations to the NCBI database format. We have confirmed that all weblinks were still current.

We also updated citations from preprints where those articles are now published.

2. Please consistently define abbreviations in tables and figures e.g., USD, DALYS

We now use $ to indicate US dollars throughout, and annotate that $ means US dollars in the captions. We also include the full term for Disability Adjusted Life Years (DALYs) in every relevant table and figure caption title.
