# Supplementary material for: COVID-19 vaccination in Sindh Province, Pakistan: A modelling study of health impact and cost-effectiveness
Source: PLoS Med. 2021 Oct 4;18(10):e1003815. doi: 10.1371/journal.pmed.1003815 (PMC8523052; doi:10.1371/journal.pmed.1003815)
Supplement: Attachment — Submitted filename: PlosMed VAC response.docx [file pmed.1003815.s003.docx]

**Review of Manuscript PMEDICINE-D-21-00936R1**

**“Health impact and cost-effectiveness of COVID-19 vaccination in Sindh Province, Pakistan”**

**Response to Authors – 11 June 2021**

**Minor Essential Revisions:**

Happy with the responses and revisions that the authors have made to their manuscript. All revisions add value and necessary clarity.

**Methods:**

The authors have made clarifying adjustments to their Table 1. I would suggest the following additional adjustments:

- Include a vaccine price of $6 in price range as this is used in Figure 4.
- Regarding vaccine wastage, Page 7 of the SI should read “times 85% (for 15% wastage)”, not 90%. Also, should this not be updated in Table 1? Wastage is 15% of vaccine procurement price per dose (not 10%)?
- Duration of vaccine-induced immunity. In the results look at 1, 2.5., 5 and life-long. In Table 1, don’t mention 5 year.
- The authors have added a lot more information regarding the costing to Table 1. Thank you, it is clearer. However, I think it would still be worthwhile having sub-totals showing the cost of delivery per dose that is mentioned on page 14 as $1.01 as a quick calculation that I did I could not replicate this figure. In the SI, it states that the cost of an AD syringe and safety box is also included. Is this included in the $3 or not. It might account for my inability to replicate the cost.

**Results**

Thank you for adding the additional scenarios – namely, slow and fast roll-out. I am surprised that less deaths are averted in the Fast roll-out scenario relative to the Slow roll-out. This would suggest that there are benefits to a slow roll-out – cheaper and averts more deaths? Surely there are more benefits to vaccinating the population quickly in a short space of time? Or is this mitigated by the vaccine-induced immunity of 2.5 years. I am also surprised that the 1 dose regimen is cost-saving. Is this because it is essentially half the price per vaccine (e.g. $1.5)? That does not really make sense.
